# Supplementary material for: MG53 suppresses interferon-β and inflammation via regulation of ryanodine receptor-mediated intracellular calcium signaling
Source: Nat Commun. 2020 Jul 17;11:3624. doi: 10.1038/s41467-020-17177-6 (PMC7368064; doi:10.1038/s41467-020-17177-6)
Supplement: Supplementary file 1 — Supplementary Information [file 41467_2020_17177_MOESM1_ESM.pdf]

MG53 suppresses interferon-β and inflammation via regulation of ryanodine receptor-mediated intracellular calcium signaling

Supplementary Information

Sermersheim et.al.

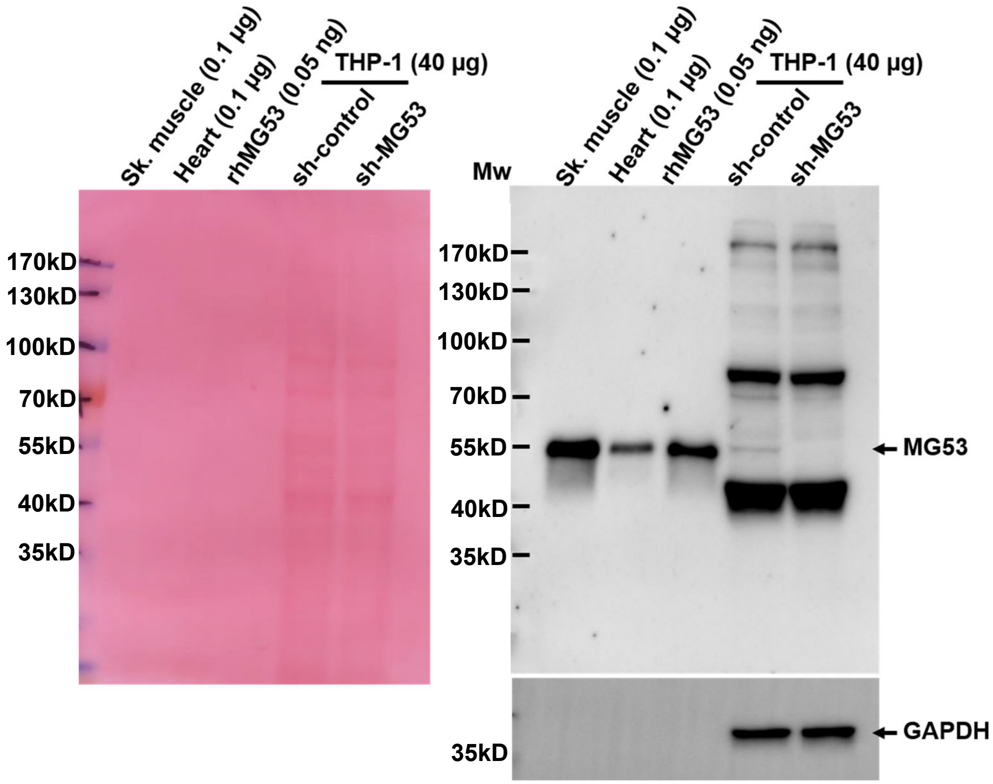

Supplementary Figure 1. Low level of MG53 expression in THP1 cells.
Mouse skeletal muscle (0.1 µg, *lane 1*), mouse heart (0.1 µg, *lane 2*), THP1 sh-control (40 µg, lane 4) and THP1 sh-MG53 (40 µg, lane 5) lysates were loaded onto a SDS-PAGE gel and probed with a custom-made rabbit monoclonal antibody against MG53. rhMG53 (0.05 ng, lane 3) was used as reference standard. Sh-MG53 cell lysate was used as a negative control. GAPDH was shown as protein loading control. MW- molecular weight markers. Left panel show Ponceau S stain of the gel. Data is representative of 4 independent experiments.

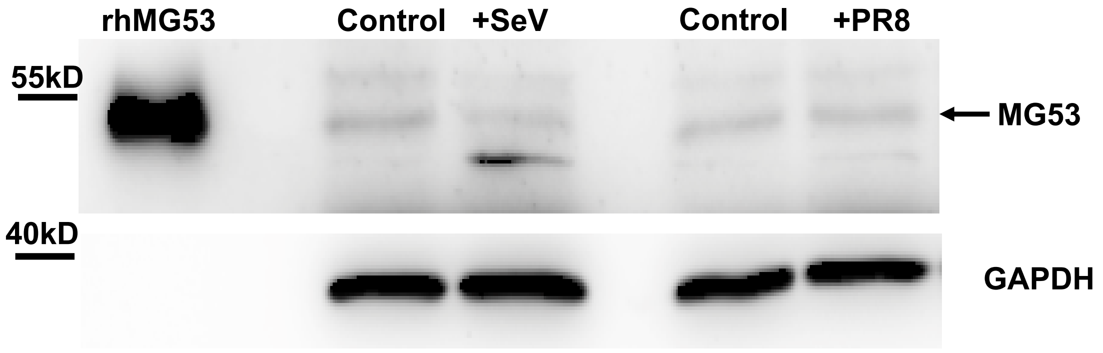

Supplementary Figure 2. Comparative effect of Sev and PR8 infection on MG53 expression in THP1 cells.

THP1 cells were infected with Sev (MOI = 5) or PR8 (MOI = 5) for 24 hours. 30 μg total cell lysate protein was loaded per lane. Western blot was performed with custom-made rabbit monoclonal antibody against MG53. Data is representative of 4 other experiments.

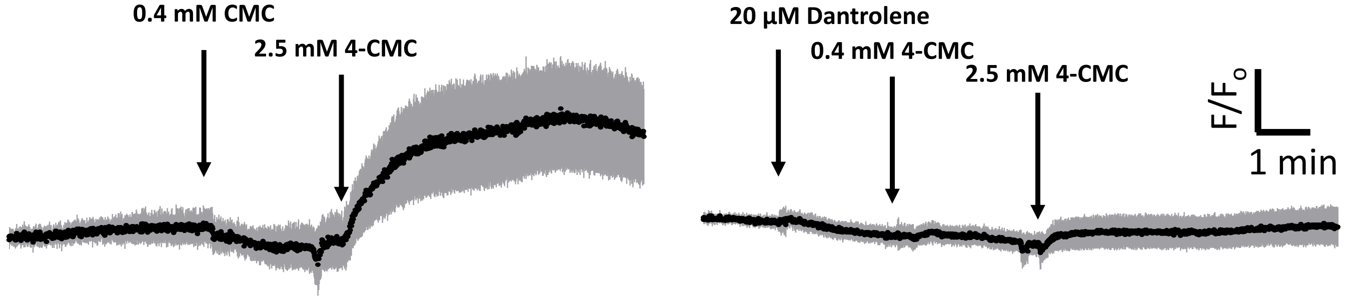

Supplementary Figure 3. Dantrolene inhibits 4-CMC induced calcium release from THP1 cells. THP1 cells were loaded with 2.5 µM Flou-4-AM for 45 mins at 37º in a 5% CO_2 incubator. 2.5 mM 4-CmC induced intracellular calcium release in THP-1 cells (extracellular solution containing 0 Ca^{2+} plus 0.5 mM EGTA), while 0.4 mM 4-CmC was insufficient. When THP1 cells were treated with 20 µM dantrolene, 4-CmC no longer was be able to trigger intracellular calcium release.

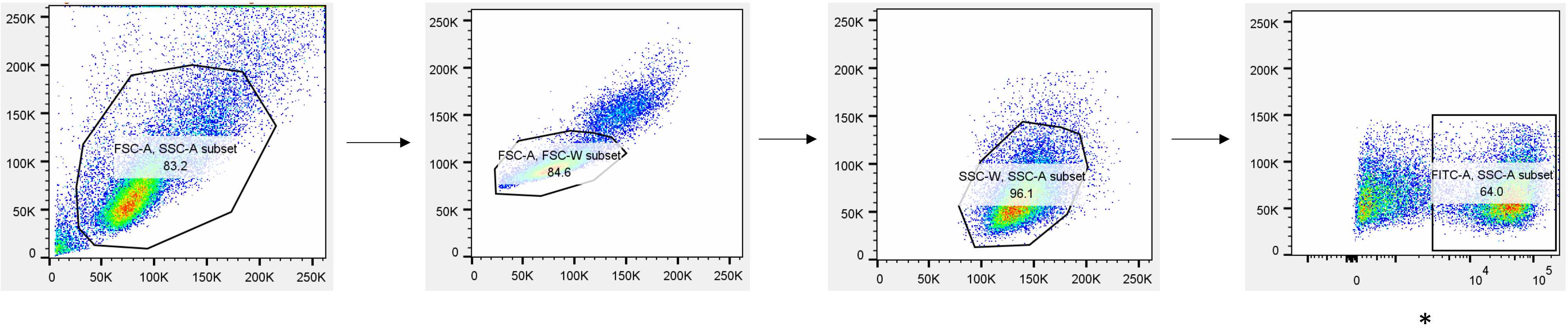

Supplementary Figure 4. Gating Strategy for detection of SeV-GFP infected THP1 cells.
All singlet cells were analyzed. Gating was used to eliminate debris and multiplet cells using forward and side scatter parameters. Gating for infected cells was based on a lack of positive cells in these gates in the non-infected control samples.* denotes gating panel that corresponds to data seen in figure 2b.
