# Supplementary material for: MG53 suppresses interferon-β and inflammation via regulation of ryanodine receptor-mediated intracellular calcium signaling
Source: Nat Commun. 2020 Jul 17;11:3624. doi: 10.1038/s41467-020-17177-6 (PMC7368064; doi:10.1038/s41467-020-17177-6)
Supplement: Supplementary file 3 — Description of Additional Supplementary Information [file 41467_2020_17177_MOESM3_ESM.pdf]

Description of Additional Supplementary Files

File Name: Supplementary Movie 1 and Supplementary Movie 2

Description: sh-control and sh-MG53 THP1 cells were PMA differentiated into macrophages and loaded with Fluo4-AM for detection of spontaneous calcium oscillations. Fluo4 fluorescence was imaged on a Nikon A1 microscope at a frame rate of 30 frames per second (fps). Compared with sh-control, sh-MG53 THP1 cells show increased frequency of spontaneous calcium oscillations.
