# Supplementary material for: MG53 suppresses interferon-β and inflammation via regulation of ryanodine receptor-mediated intracellular calcium signaling
Source: Nat Commun. 2020 Jul 17;11:3624. doi: 10.1038/s41467-020-17177-6 (PMC7368064; doi:10.1038/s41467-020-17177-6)
Supplement: Supplementary file 6 — Reporting Summary [file 41467_2020_17177_MOESM6_ESM.pdf]

nature research

Corresponding author(s): Jianjie Ma and Jacob S. Yount

Last updated by author(s): 04/29/2020

Reporting Summary

Nature Research wishes to improve the reproducibility of the work that we publish. This form provides structure for consistency and transparency in reporting. For further information on Nature Research policies, see Authors & Referees and the Editorial Policy Checklist .

Statistics

For all statistical analyses, confirm that the following items are present in the figure legend, table legend, main text, or Methods section.

n/a	Confirmed	
☐	☒	The exact sample size (*n*) for each experimental group/condition, given as a discrete number and unit of measurement
☐	☒	A statement on whether measurements were taken from distinct samples or whether the same sample was measured repeatedly
☐	☒	The statistical test(s) used AND whether they are one- or two-sided<br>*Only common tests should be described solely by name; describe more complex techniques in the Methods section.*
☐	☒	A description of all covariates tested
☐	☒	A description of any assumptions or corrections, such as tests of normality and adjustment for multiple comparisons
☐	☒	A full description of the statistical parameters including central tendency (e.g. means) or other basic estimates (e.g. regression coefficient) AND variation (e.g. standard deviation) or associated estimates of uncertainty (e.g. confidence intervals)
☐	☒	For null hypothesis testing, the test statistic (e.g. *F*, *t*, *r*) with confidence intervals, effect sizes, degrees of freedom and *P* value noted<br>*Give P values as exact values whenever suitable.*
☒	☐	For Bayesian analysis, information on the choice of priors and Markov chain Monte Carlo settings
☒	☐	For hierarchical and complex designs, identification of the appropriate level for tests and full reporting of outcomes
☒	☐	Estimates of effect sizes (e.g. Cohen's *d*, Pearson's *r*), indicating how they were calculated

Our web collection on statistics for biologists contains articles on many of the points above.

Software and code

Policy information about availability of computer code

Data collection	SoftmaxPro, CellSens Dimension, FACSCanto II with FACSDiva software, FlowJo, Veritas Microplate Luminometer, CFX-Maestro, ZEN2011 SP2 Black edition software, NiKon A1R, and Photon Technology International
Data analysis	Graphpad Prism 8

For manuscripts utilizing custom algorithms or software that are central to the research but not yet described in published literature, software must be made available to editors/reviewers. We strongly encourage code deposition in a community repository (e.g. GitHub). See the Nature Research guidelines for submitting code & software for further information.

Data

Policy information about availability of data

All manuscripts must include a data availability statement. This statement should provide the following information, where applicable:

- Accession codes, unique identifiers, or web links for publicly available datasets
- A list of figures that have associated raw data
- A description of any restrictions on data availability

The authors declare that all data supporting the findings of this study are available within the paper and its supplementary information files.

Field-specific reporting

Please select the one below that is the best fit for your research. If you are not sure, read the appropriate sections before making your selection.

☒ Life sciences ☐ Behavioural & social sciences ☐ Ecological, evolutionary & environmental sciences

Life sciences study design

All studies must disclose on these points even when the disclosure is negative.

Sample size	Groups of at least five mice were utilized for most experiments based on our historical experience and published literature regarding numbers needed to observe statistically significant differences in weight loss, survival, viral titers, and cytokine measurements in influenza virus infections.
Data exclusions	No data were excluded.
Replication	The biochemical and molecular biology experiments in this paper have mostly been repeated at least 2 times (total 3 independent experiments). We didn't notice failure of the replication experiments.
Randomization	Randomization was not required. All WT and MG53 KO mice in our experiments were infected.
Blinding	Daily weighing of the mice was not done in a blinded fashion. Virus titering and cytokine measurements from in vivo samples were performed in a blinded manner without knowledge of sample identity. Analysis of flow cytometry was performed by a scientist aware of whether samples were infected or not, but blind to genetic background. Remaining experiments were performed by singular individuals who were ergo not blinded during data collection or analysis.

Reporting for specific materials, systems and methods

We require information from authors about some types of materials, experimental systems and methods used in many studies. Here, indicate whether each material, system or method listed is relevant to your study. If you are not sure if a list item applies to your research, read the appropriate section before selecting a response.

Materials & experimental systems

n/a	Involved in the study	
☐	☒	Antibodies
☐	☒	Eukaryotic cell lines
☒	☐	Palaeontology
☐	☒	Animals and other organisms
☐	☒	Human research participants
☒	☐	Clinical data

Methods

n/a	Involved in the study	
☒	☐	ChIP-seq
☐	☒	Flow cytometry
☒	☐	MRI-based neuroimaging

Antibodies

Antibodies used	MG53 (custom rabbit monoclonal antibody), GAPDH (CST 2118), p65 (CST 8242), phospho-p65 (CST 3033), and RyR (Invitrogen MA3925), CD45, Abcam ab10558
Validation	Custom-made anti-MG53 antibody is validated with tissues from WT and mg53-/- tissues. This antibody has also been used and validated by other investigators. We validated the antibodies based on molecular weight, reports on the manufacture websites and published research articles that cited the same source of the antibodies as we used in our study. All Cell Signaling Technologies antibodies are made to adhere to the Hallmarks of Antibody Validation (https://www.cellsignal.com/contents/our-approach/cst-antibody-validation-principles/ourapproach-validation-principles). CST2118 has been validated by the CST for WB, IHC, IF, and F and has been cited 3075 times. CST8242 has been validated by CST for WB, IP, IHC, IF, F, and ChIP and has been cited 1339 times. CST3033 has been validated by CST for WB, IP, IF, and F and has been cited 1223 times. Invitrogen MA3925 has been successfully validated for WB, IHC, IF, and IP and has been cited 179 times. Ab10558 has been successfully validated for the use in Flow Cyt, IHC-Fr, IHC-P, WB, and ICC/IF and has been cited in 161 publicaitons.

Eukaryotic cell lines

Policy information about cell lines

Cell line source(s)	THP-1, LLCMK2, MDCK, and HEK293 cell lines were low passage lines received from the ATCC. HEK293FT cells were acquired from Thermo Fisher Scientific. Doxycycline inducible HEK293-RyR2 cells were graciously provided by Dr. Wayne Chen.
Authentication	ATCC derived cells have been thoroughly tested and authenticated according the manufacturer. Doxycycline inducible HEK293-RyR2 cells were authenticated by both WB/protein detection and calcium mobilization imaging following doxycycline treatment.
Mycoplasma contamination	Cell lines were not tested for mycoplasma contamination.

Commonly misidentified lines (See ICLAC register)	To our knowledge no commonly misidentified cell lines were used during these studies.

Animals and other organisms

Policy information about studies involving animals; ARRIVE guidelines recommended for reporting animal research

Laboratory animals	Species: Mus musculus, Strain: 129S1/SvlmJ , Sex: Male, Age: 12 weeks.MG53 knockout mice were generated in the 129S1/SvlmJ strain of mice. Mice have been backcrossed and maintained for over 30 generations and used in numerous tissue injury studies. All mice were housed and handled in the Ohio State University Laboratory Animal Resources vivarium, with temperature, humidity, and light/dark schedule controlled following Ohio State University Institutional Animal Care and Use Committee approved protocols in accord with National Institute of Health guidelines
Wild animals	The study did not use wild animals.
Field-collected samples	This study did not use samples collected in the field.
Ethics oversight	All mice were housed and handled following IACUC approved protocols. The Ohio State University IACUC protocol number is 2016A00000051.

Note that full information on the approval of the study protocol must also be provided in the manuscript.

Human research participants

Policy information about studies involving human research participants

Population characteristics	Not applicable
Recruitment	Not applicable
Ethics oversight	Western Blot shown in Figure 1B was obtained via protein lysates from de-identified human cells.

Note that full information on the approval of the study protocol must also be provided in the manuscript.

Flow Cytometry

Plots

Confirm that:

- [x] The axis labels state the marker and fluorochrome used (e.g. CD4-FITC).
- [x] The axis scales are clearly visible. Include numbers along axes only for bottom left plot of group (a 'group' is an analysis of identical markers).
- [x] All plots are contour plots with outliers or pseudocolor plots.
- [x] A numerical value for number of cells or percentage (with statistics) is provided.

Methodology

Sample preparation	THP1 cells were washed in PBS and then fixed in 4% PFA. Samples were then washed and resuspended in PBS.
Instrument	FACSCanto II flow cytometer (BD Biosciences)
Software	Data were collected using FACSDiva (BD) and analyzed using FACSDiva (BD) or FlowJo v7.6 or v10. Graphing and statistical analysis were performed using Prism 8 (GraphPad).
Cell population abundance	We did not sort our cells in our experiments.
Gating strategy	All singlet cells were analyzed. Gating was used to eliminate debris and multiplet cells using forward and side scatter parameters. Gating for infected cells was based on a lack of positive cells in these gates in the non-infected control samples.

- [x] Tick this box to confirm that a figure exemplifying the gating strategy is provided in the Supplementary Information.
